# Antimicrobial Peptides: Virulence and Resistance Modulation in Gram-Negative Bacteria

**DOI:** 10.3390/microorganisms8020280

**Published:** 2020-02-19

**Authors:** Marylise Duperthuy

**Affiliations:** Département de Microbiologie, Infectiologie et Immunologie, Université de Montréal, Succ. Centre-ville, Montréal, QC H3C 3J7, Canada; marylise.duperthuy@umontreal.ca

**Keywords:** antimicrobial peptide, subinhibitory concentration, Gram-negative, virulence, resistance

## Abstract

Growing resistance to antibiotics is one of the biggest threats to human health. One of the possibilities to overcome this resistance is to use and develop alternative molecules such as antimicrobial peptides (AMPs). However, an increasing number of studies have shown that bacterial resistance to AMPs does exist. Since AMPs are immunity molecules, it is important to ensure that their potential therapeutic use is not harmful in the long term. Recently, several studies have focused on the adaptation of Gram-negative bacteria to subinhibitory concentrations of AMPs. Such concentrations are commonly found in vivo and in the environment. It is therefore necessary to understand how bacteria detect and respond to low concentrations of AMPs. This review focuses on recent findings regarding the impact of subinhibitory concentrations of AMPs on the modulation of virulence and resistance in Gram-negative bacteria.

## 1. Introduction

Antimicrobial resistance is one of the most significant public health threats that the world is facing today. Resistance to antibiotics increases health costs, severity of infections, and death rates. It has been estimated that 10 million premature deaths will occur by 2050 due to antimicrobial resistance, with a cumulative cost of US$100 trillion [1]. Even though these figures have been criticized, there is a consensus on the urgent need to develop alternative treatments to counteract the antibiotic resistance crisis [2,3]. The alternatives can be classified in two groups: (i) drugs that can kill bacteria, including new antimicrobials or phages, and (ii) drugs that can disarm the pathogens, such as anti-virulence or anti-signaling molecules. Among all challenges of the bacterial resistance, Gram-negative pathogens are particularly worrisome, because they are becoming resistant to nearly all antimicrobial drugs [4].

One of the proposed alternatives to antibiotics are antimicrobial peptides (AMPs). AMPs are ubiquitous oligopeptides, often cationic and amphipathic, a property that facilitates their interaction with the anionic charge of bacterial membranes and their integration into these hydrophobic membranes. Antimicrobial peptides are usually categorized according to their structure: α-helical, β-sheet, extended, or loop. At a lethal concentration, AMPs can form pores in the bacterial membrane, leading to the leakage of periplasmic and cytoplasmic content and to the death of the cells. Alternatively, AMPs can cross the bacterial membrane and interfere with intracellular components involved in nucleic acid, protein, or cell wall synthesis. The mechanisms of action of AMPs have already been extensively reviewed elsewhere [5,6]. Because of their multiple targets in the bacterial cells, it was expected that the risk of developing resistance by bacterial pathogens is low. However, several studies demonstrated otherwise. In Gram-negative bacteria, the resistance to AMPs involves modifications of the lipopolysaccharides (LPS) on the outer membrane, modifications of the phospholipids within the inner membrane, activation of efflux pumps, trapping of AMPs by a capsule or by membrane vesicles, and proteolytic degradation of AMPs (for review: [7]).

Besides their antimicrobial activities, AMPs also display immunomodulatory functions including the induction or the modulation of cytokine and chemokine production, the inhibition of pro-inflammatory pathways, the modulation of host gene expression, or the chemo-attraction of immune cells (for review: [8]). At a low concentration, AMPs can also induce the modulation of the expression and secretion of virulence, resistance, or colonization effectors by bacterial pathogens. This review will focus on the modulation of the expression of genes and resistance or virulence effectors from Gram-negative bacteria in the presence of sublethal concentrations of AMPs.

## 2. Global Response to AMPs in Gram-Negative Bacteria

Most of the works addressing the effects of sublethal concentrations of AMPs on Gram-negative bacteria are global transcriptomic and proteomic studies. In all these studies, many genes or proteins are deregulated in the presence of AMPs, which reflects the active response of bacteria to the stress induced by AMPs. Among these genes or proteins, some are involved in resistance or in virulence and might reflect a specific response of the bacteria to AMPs. For instance, a microarray analysis of *Neisseria meningitidis* demonstrated that the expression of more than 200 genes was modified after a 1 h of contact with a sublethal concentration of LL-37, including the genes encoding for capsule polysaccharides, a major virulence factor of this pathogen [9]. In *Pseudomonas aeruginosa*, a microarray analysis demonstrated that 420 genes were deregulated in the presence of a sublethal concentration of LL-37, including genes involved in quorum sensing, virulence, and resistance [10]. In *Escherichia coli*, 175 genes were differentially expressed in the presence of ApoEdpL-W, an AMP derived from human apolipoprotein E, some of them being involved in stress response, iron acquisition, and membrane synthesis [11]. Besides transcriptomic analyses, global proteomic profiles have also been studied in the presence of sublethal concentrations of AMPs. In *Salmonella enterica* serovar Typhimurium, a bi-dimensional analysis of total proteins demonstrated that six proteins were more abundant and one protein was less abundant in the presence of the human Bactericidal Permeability Protein (BPI), a protein with antimicrobial activity [12]. However, only one protein was affected in the presence of polymyxin B, suggesting that the bacterial response to sublethal concentrations of AMPs is AMP-dependent [12]. In *Clostridium difficile*, a quantitative mass spectrometry analysis identified 61 and 54 proteins for which the abundance differed in the strains 630 and 6477, respectively, in the presence of a sublethal concentration of LL-37 [13]. Among them, only 16 were common to both strains, indicating a strain-dependent response to sublethal concentrations of AMPs. Altogether, these studies demonstrate that Gram-negative bacteria can detect the presence of subinhibitory concentrations of AMPs and globally respond by modulating the expression and secretion of series of genes and proteins including virulence and resistance effectors.

## 3. Resistance Modulation

### 3.1. Effect of a Pre-Challenge with AMPs on Resistance

An increasing number of studies demonstrated that a pre-challenge with sublethal concentrations of AMPs can provide protection against AMPs. This effect is usually lost after several rounds of freezing and growing, indicating an inducible mechanism. Thus, a pre-incubation of *Klebsiella pneumoniae* with polymyxin B resulted in increased resistance to polymyxin B and cross-resistance to other AMPs such as the human defensins hBD1, hBD2, and HNP1 and magainin 2 [14]. This increased resistance is partly due to the upregulation of the *cps* genes coding for capsule polysaccharides (CPS), which are released in the environment and act as a shield, trapping the AMPs before they can reach the bacterial cells [14,15]. Similarly, an up-regulation of the capsule constituents has also been observed in *N. meningitidis* in the presence of a sublethal concentration of LL-37 [9]. In addition to the induction of *cps* expression, in *K. pneumoniae*, a modification of LPS with the addition of aminoarabinose and palmitate has also been observed and might contribute to the protective effect in *K. pneumoniae* observed after a pre-incubation with polymyxin B [14]. In *Neisseria gonorrhoeae*, a pre-incubation with a sublethal concentration of polymyxin B increased the resistance to this AMP. The mechanism behind this protective effect is dependent on MisR, a global transcriptional regulator controlling membrane integrity [16].

A pre-challenge with AMPs can also increase the resistance to antibiotics. In *P. aeruginosa*, the resistance to fluoroquinolone and aminoglycoside increased following a pre-incubation with the human cathelicidin LL-37 [10]. The authors suggested that the increased resistance to the antibiotics was due to an upregulation of efflux pumps and to membrane modifications involved in antibiotic resistance [10]. Similar cross-resistance observations have been reported for *Streptococcus pneumoniae*, a Gram-positive pathogen [17]. Since AMPs are usually produced at the site of infection, these cross-resistance mechanisms are particularly worrisome. Indeed, antibiotic resistance might be more severe at the site of infection than predicted *in vitro*. Cross-resistance mechanisms have also been reported between different AMPs in several Gram-negative bacteria [18,19,20]. Conversely, synergistic effects between AMPs and antibiotics, as well as therapies combining these antimicrobial compounds, have been proposed [21]. However, the synergy and cross-resistance mechanisms between AMPs and between AMPs and antibiotics are still poorly understood. Identifying these mechanisms would be of great interest for the development of AMP–antibiotic dual therapies and for the prediction of the repercussions of using an exogenous antimicrobial cocktail on the efficacy of the AMP produced by the host at the site of infection.

### 3.2. Activation of Signaling Pathways Leading to Resistance

The detection of AMPs by bacteria usually involves sensor proteins located at the bacterial surface, i.e., in the membrane. Among these signal detection systems, the PhoP/PhoQ two-component system of *Salmonella* has been widely studied for its implication in AMP detection and resistance. PhoQ is a sensor histidine kinase activated by phosphorylation in the presence of AMPs. Following the activation of PhoQ, the transcriptional regulator PhoP is activated by transfer of the phosphate from the conserved histidine residue of PhoQ. Activation of PhoP will result in the binding of PhoP to target DNA sequences and modulation of the expression of specific genes. Among those genes, *pagL* is specific to *Salmonella* and is involved in the deacylation of lipid A. The expression of *pmrD* is also induced by *phoP* in the presence of subinhibitory concentrations of AMPs. PmrD is an activator of PmrA, the transcriptional response regulator of the PmrA/PmrB two-component system. The activation of PmrA also leads to the expression of genes involved in LPS modification and AMP resistance, including the *arnBCADTEF* and *pmrCAB* operons and the genes *cptA* and *pmrE*. Altogether, these genes are involved in the modification of LPS by the addition of positively charged arabinosamines, which reduce the anionic charge of the membrane and decrease the interaction with the AMPs [22], and of phosphoethanolamines which promote a conformational rearrangement of LPS [23], leading to increased resistance to AMPs. Two-component systems activated by sublethal concentrations of AMPs have been described in other Gram-negative bacteria, e.g., the CprRS [24] and PmrAB [22] systems of *P. aeruginosa*, the CpxRA system of *E. coli* [25], and the MirRS system of *N. meningitidis* [26]. In regard to the number of potential two-component systems annotated in the genomes of Gram-negative bacteria, it is likely that some of them respond to the presence of sublethal concentrations of AMPs in order to promote resistance.

Besides the two-component system, porins, located in the outer membrane of Gram-negative bacteria are also involved in detecting AMPs in order to activate resistance mechanisms. For instance, in *Vibrio cholerae*, the OmpU porin serves as a sensor for membranolytic AMPs [27,28]. In the presence of AMPs, OmpU is destabilized and exposes a YDF motif in the periplasm, leading to the activation of the DegS protease. Subsequently, DegS cleaves RseA, the inner membrane anchoring system for the alternative sigma factor E, while the liberated sigmaE induces the expression of genes involved in LPS modification and AMP resistance [27,28].

### 3.3. Induction of AMP Trapping Mechanisms

Several bacteria have developed entrapping mechanisms of AMPs for resistance, which limit the concentration of AMPs reaching the bacterial cell. Therefore, because of AMPs dilution, the impact of AMPs on the bacteria is reduced. In *V. cholerae*, we demonstrated that growing the bacteria with polymyxin B produced membrane vesicles with a bigger size and a modified protein profile, which conferred cross-resistance to LL-37 [18]. This cross-resistance was mediated by the trapping of LL-37 by the biofilm matrix protein Bap1 associated with OmpT at the surface of the membrane vesicles of *V. cholerae* only when the bacteria were grown in the presence of polymyxin B [18]. In another *Vibrio* species, *Vibrio tasmaniensis*, we observed a similar effect. The membrane vesicles of *V. tasmaniensis* can trap polymyxin B in a dose-dependent manner, which confers resistance by a dilution effect [29]. Similarly, the membrane vesicles of *E. coli* can trap polymyxin B, and incubation of *E. coli* with a sublethal concentration of polymyxin B induced the massive release of membrane vesicles, conferring higher resistance to polymyxin B [30].

Some Gram-negative bacteria express a surface capsule composed of polysaccharides. Since the capsule is anionic, it can trap cationic AMPs, leading to the inactivation of their antimicrobial activity and to increased bacterial resistance [15]. The expression of capsule biosynthesis genes can be activated in the presence of AMPs. For instance, in *K. pneumoniae*, the expression of the *cps* operon is activated in the presence of polymyxin B and enhances resistance to this AMP [31]. The authors of this study also reported a positive correlation between the quantity of CPS and the resistance to polymyxin B [31]. Similarly, in *N. meningitidis*, the presence of the capsule protects the bacteria against various AMPs, including defensins, cathelicidins, protegrins, and polymyxin B [32]. The release of the capsule in *N. meningitidis* increases the resistance to human cathelicidin LL-37 [9], and subinhibitory concentrations of AMPs induce the expression of the capsule biosynthesis genes [9,32].

An indirect trapping of AMPs by the host cells and induced by *P. aeruginosa* has been described. This mechanism involves the secretion of LasA, a virulence factor of *P. aeruginosa*, which induces the shedding of syndecan-1, one of the main human cell surface heparan sulfate proteoglycans and a receptor of extracellular ligands [33]. The syndecan-1 ectodomain generated from the shedding can trap cathelicidins rich in proline and arginine and inhibit their antimicrobial activity [34]. The shedding of syndecan-1 by LasA of *P. aeruginosa* is enhanced in vivo in the lung, an environment rich in cationic AMPs [34]. Whether the increased activation of the shedding process is due to an increased secretion of LasA as well as the role of the AMPs in this process remain to be determined.

### 3.4. Induction of Proteases

Another recognized mechanism of AMP resistance is the extracellular degradation of AMPs by secretion of proteases. In *S. enterica*, the PgtE protease is involved in the proteolytic degradation of AMPs [35]. The regulation of PgtE is mediated by PhoPQ, a two-component system activated in the presence of AMPs (see section: *Activation of signaling pathways leading to resistance*), and the abundance of PgtE is enhanced upon PhoP activation. PgtE belongs to the Omptin family, a group of aspartate proteases located in the outer membrane of enterobacteria, which includes OmpT from *E. coli* and Pla from *Yersinia pestis* [35]. Proteases from this family cleave AMPs with α-helical structure only, such as LL-37 [36,37].

Other proteases belonging to the metalloprotease family are also involved in AMP resistance. The metalloproteases ZmpA and ZmpB from *Burkholderia cenocepacia* can cleave various AMPs, but only ZmpA cleaves the linear LL-37, and only ZmpB cleaves the nonlinear HBD-1 [38]. However, the expression of these metalloproteases was not modified in the presence of sublethal concentrations of polymyxin B [39], which might be explained by the major structural differences between polymyxin B and the AMPs targeted by ZmpA and ZmpB. In *V. cholerae*, we reported the activation of the secretion of PrtV in the presence of sublethal concentrations of cathelicidin LL-37 [40]. Even though no deleterious effect on AMP resistance has been demonstrated upon *prtV* mutation, the bacteria sur-expressing PrtV are more resistant to LL-37 [40]. PrtV is a metalloprotease with high homology to InhA of *Bacillus* [41], which degrades cecropin A [42], an AMP that shares a similar helical structure with LL-37. Therefore, it is reasonable to hypothesize that the resistance mechanism mediated by PrtV involves the proteolytic degradation of LL-37.

### 3.5. Transport Systems

Membrane transport systems are key effectors of antimicrobial resistance. Efflux systems are especially important for antibiotic resistance [43,44,45], and drugs inhibiting these systems represent promising alternatives to antibiotics [46,47,48,49]. Similarly, efflux pumps can be involved in AMP resistance [50]. In *Yersinia enterocolitica*, the temperature-regulated efflux pump/potassium antiporter RosA/RosB, is involved in antibiotic and AMP resistance [51]. The mechanism seems to involve the efflux of AMPs by RosA after they enter the cytoplasm, using the energy provided by RosB. The expression of the *ros* locus encoding for the RosA/RosB system increases with temperature and in the presence of subinhibitory concentrations of polymyxin B [51], demonstrating the ability of the efflux pump/potassium antiporter system to activate the resistance mechanism on demand. Other efflux pumps with a role in AMP resistance in Gram-negative pathogens have been reported in *Vibrio*, *Salmonella*, *Klebsiella*, and *Neisseria*, but their modulation by AMPs remains to be determined [52,53,54,55,56].

An unexpected resistance mechanism involving the influx of AMPs has been described in non-typeable *Haemophilus influenzae*. This mechanism involves the uptake of AMPs through the *sap* (sensibility to antimicrobial peptides) operon, which encodes ABC transporter proteins including the periplasmic substrate-binding protein SapA, SapD and SapF ATPases, and SapB and SapC permeases [57,58]. Once inside the bacterial cell, AMPs are degraded by cytoplasmic proteases [58]. The authors propose that the Sap import system is important to limit the accumulation of AMPs, such as the host cathelicidin LL-37 and defensin hBD3, in the membrane and in the periplasm, therefore limiting their lethal pore-forming effect [58]. Finally, *sapA* gene expression is increased in the middle ear of chinchilla, in a model of otitis media caused by non-typeable *H. influenzae* [59]. The chinchilla and the human middle ear contain AMPs, especially β-defensins [60,61]. Due to the sensitivity of the middle ear pathogens to β-defensins [62], it has been proposed that this AMP plays a key role in the defense against pathogens. However, there is currently no evidence that AMPs are involved in the upregulation of the *sap* operon.

### 3.6. Modulation of Biofilm Formation

A significant part of antimicrobial resistance is associated with biofilms. Biofilms are structured bacterial communities embedded within an extracellular matrix that protects them from environmental stressors, including antimicrobials. Bacterial biofilms represent a major public health problem because they are up to 1000 times more resistant to antimicrobial agents than the planktonic form and often lead to therapy failure. Therefore, biofilms are often associated with pathogen resistance and persistence inside the host. Similarly, in the environment, bacteria organized in biofilms display a better survival than their planktonic counterparts. The effects of subinhibitory concentrations of antimicrobial peptides on biofilm formation have been studied, and both positive and negative effects have been reported.

Pro-biofilm effects of subinhibitory concentrations of AMPs have been reported for Gram-negative bacteria. For instance, biofilm formation is activated by polymyxin B and colistin in *Acinetobacter baumanii*, an opportunistic pathogen often associated with nosocomial infections [63]. Conversely, inhibition of biofilm formation by cationic AMPs has been reported. A decrease in biofilm formation has been observed in the presence of subinhibitory concentrations of several synthetic antimicrobial peptides for *P. aeruginosa* and *Burkholderia cenocepacia* [64]. Similarly, a reduction in biofilm formation has been reported in *P. aeruginosa* in the presence of polymyxin B, but the mechanism of biofilm inhibition remains to be elucidated [65]. This reduction is accentuated by the combination of polymyxin B with gramicin S, another AMP. We recently demonstrated that a subinhibitory concentration of PmB can inhibit biofilm formation in *V. cholerae* [66]. This inhibition occurred during the early stage of biofilm formation and was correlated with a reduction in the number of flagellated bacteria. Therefore, besides biofilm formation, *V. cholerae* motility was also impaired in the presence of PmB [66]. A similar effect of indolicin has been reported on *P. aeruginosa* motility and biofilm formation and involves PsrA, a positive regulator of the type III secretion system important for virulence [67,68].

## 4. Virulence Modulation

A role for AMPs in the modulation of pathogens’ virulence has been reported. This modulation usually occurs by interference with the signaling pathways leading to virulence expression, such as the quorum sensing pathway. This modulation can lead to the regulation of the expression and secretion of virulence factors or to a missed communication between cells. In both cases, virulence can be affected, either positively or negatively.

### 4.1. Quorum-Sensing Interference

In many Gram-negative bacteria, the expression of virulence factors is under the control of quorum sensing, a system dependent on bacterial density. It has been demonstrated that some AMPs can interfere with quorum sensing by a mechanism known as quorum quenching. The production of violacein by *Chromobacterium voilaceum* is quorum-sensing-dependent and is inhibited in the presence of subinhibitory concentrations of subtolisin A, an AMP produced by *Bacillus subtilis* [69]. In this study, the authors also demonstrated that *E. coli* biofilm formation was inhibited in the presence of subtolisin A [69]. Since quorum sensing is essential for inter-bacterial communication and virulence expression in several pathogenic bacteria, the ability of AMPs to act as quorum-quenching molecules is of great interest in the development of anti-biofilm drugs.

Conversely, some AMPs can act as quorum-sensing activators. Indeed, a subinhibitory concentration of colistin induced the upregulation of the *Pseudomonas* quinolone signal (PQS) genes [70]. It has been proposed that the PQS molecules are associated with the membrane lipids and are therefore transported from cell to cell by membrane vesicles [71]. Since the outer membrane is a target of colistin, the release of PQS molecules might increase in the presence of subinhibitory concentrations of colistin, especially the PQS contained in the membrane vesicles. Therefore, the effect of colistin as an activator of quorum sensing is dual, *i.e*., it increases the expression of the PQS genes and it increases the release of PQS in the extracellular environment by membrane vesicles.

### 4.2. Virulence Factor Production

The presence of subinhibitory concentrations of AMPs in the bacterial environment can lead to the activation of virulence factors expression and secretion. This is the case for *P. aeruginosa* in the presence of subinhibitory concentrations of LL-37 [10]. In this study, the authors demonstrated that the secretion of toxic metabolites, such as pyocyanin, elastase, the PQS system, and some proteases, was increased in the presence of LL-37. This accrued secretion might be the reflection of the overexpression of *pqsE* encoding PqsE, a major regulator of virulence controlling the production of virulence factors and required for full virulence of *P. aeruginosa* in mice [72]. Conversely, the presence of subinhibitory concentrations of synthetic AMPs with linear structure was not able to induce the secretion of virulence factors, suggesting that this response is specific to the host LL-37 or to AMPs with similar helical structure [10].

In *Klebsiella* spp., the capsule is an important effector of virulence [73]. Indeed, it has been demonstrated that virulence is correlated with the type and the amount of CPS produced by *K. pneumoniae* [74]. In addition, CPS are used as a decoy to trap and inactivate AMPs [15,31]. In *K. pneumoniae*, the presence of subinhibitory concentrations of polymyxin B or human neutrophil α-defensin 1 (HNP-1), induced the release of CPS by the bacterial membrane, which in turn increased the resistance to AMPs [15]. There is currently no evidence that the increased release of CPS in the presence of subinhibitory concentrations of AMPs is correlated with an increase in virulence. However, given the role of the CPS in virulence, it is possible that subinhibitory concentrations of AMPs have a role in the virulence potential of *K. pneumoniae.*

In *V. cholerae*, we demonstrated that subinhibitory concentrations of LL-37 induced over-secretion of PrtV associated with membrane vesicles [40]. PrtV is a protease and a virulence factor of *V. cholerae* identified in a *Caenorhabditis. elegans* model. PrtV is also essential for the survival from grazing by predators in aquatic environment [75]. However, the effect of this over-secretion on virulence remains to be established.

### 4.3. Invasion

During the infectious process, the enteric pathogen *Shigella flexneri* crosses the mucosa and adheres to epithelial cells. After adhesion, *S. flexneri* actively invades the epithelial cells and spreads to neighboring cells causing tissue damage and inflammation [76]. The adhesion is therefore essential for *S. flexneri* virulence. It has been demonstrated that subinhibitory concentrations of AMPs located in neutrophils granules and released during the infection increase the adhesion of *S. flexneri* to human cells [77,78]. Membrane-binding AMPs are usually cationic, which facilitates their interaction with the bacterial membrane. Because host cells also exhibit an anionic charge, it has been proposed that human AMPs such as the enteric α-Defensin 5 (HD-5) are used as a scaffold between the bacteria and the host cells [77,78]. In addition, a subinhibitory concentration of human defensin 5 increases the phagocytosis of *S. flexneri* by human macrophages, leading to increased cytotoxicity and lysis of the macrophages [79]. Therefore, *S. flexneri*, which is devoid of the major adhesion apparatus, subverts the human immune system and exploits AMPs’ properties to adhere and invade human epithelial cells.

## 5. Conclusions

**Figure 1:**
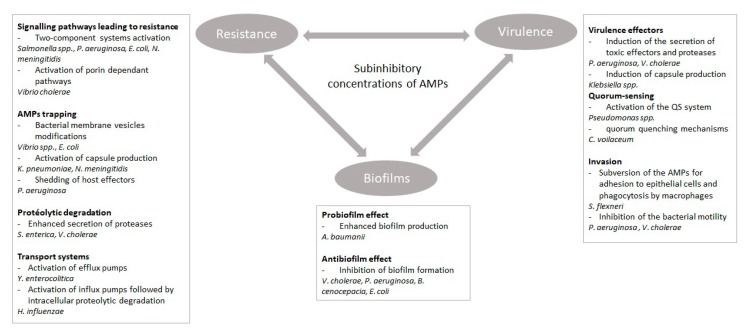
Impact of subinhibitory concentrations of antimicrobial peptides on resistance, virulence, and biofilm formation in Gram-negative bacteria. The double arrows illustrate the connections between virulence, resistance, and biofilm formation. Examples of Gram-negative bacteria responding to subinhibitory concentrations of antimicrobial peptides (AMPs) are provided in each section.

Given the evidence presented above, bacteria can detect and respond to the presence of subinhibitory concentrations of AMPs (Figure 1). Initially, it was thought that bacteria were less likely to develop resistance toward AMPs than toward antibiotics because of the multiple AMPs targets and because of the membranolytic activity of most AMPs. We now know that this assumption is not entirely true, and different mechanisms of resistance have been described [50]. In addition, membrane-independent mechanisms of action have been described, and some AMPs can kill the bacteria without altering their membranes [80]. Low concentrations of antimicrobials are usually found in the environment or in the host. Evidences suggest that these low concentrations can be used as signals for the activation of resistance mechanisms. Recent studies have examined the ability of AMPs to modulate the virulence of pathogenic bacteria. As presented in this review, several Gram-negative bacteria, including pathogenic bacteria, appear to have the ability to detect the presence of AMPs at subinhibitory concentrations. Following this detection, the production and secretion of several virulence factors can be enhanced. Conversely, some AMPs negatively impact intraspecies signaling systems, such as quorum sensing. The quorum-sensing system is often implicated in the activation of colonization or virulence and in biofilm formation by Gram-negative bacteria. From this point of view, AMPs represent a promising alternative to conventional antibiotics. Indeed, interfering with the signaling pathways allowing communication between bacteria at subinhibitory concentrations does not jeopardize their survival, thereby limiting the risk of developing resistance. However, the concentration of AMPs at the site of infection remains to be determined. Also, interactions between antimicrobials does exist, including synergistic effects, and their impact on bacterial virulence is difficult to predict. A subinhibitory concentration of a single AMP might become lethal in the presence of other AMPs, including those from the host. As a result, pathogenic bacteria might develop resistance to survive to the combined effects of several AMPs. We also demonstrated that the exogenous use of AMPs might lead to cross-resistance against host AMPs [18], which could reduce the effectiveness of the immune defenses. These elements have been very little studied to date. A deeper understanding of the consequences of using AMPs as an alternative to antibiotics or in food conservation needs to be obtained. To do this, studies aimed at determining the impact of subinhibitory concentrations of AMPs on the metabolism, physiology, and behavior of bacteria will be necessary.
